# Mannan-binding lectin in cerebrospinal fluid: a leptomeningeal protein

**DOI:** 10.1186/2045-8118-9-17

**Published:** 2012-08-13

**Authors:** Hansotto Reiber, Barbara Padilla-Docal, Jens Christian Jensenius, Alberto Juan Dorta-Contreras

**Affiliations:** 1 Neurochemistry Laboratory, University Göttingen, Göttingen, Germany; 2 Labcel, Med.Sci. University Havana, Havana, Cuba; 3 Department of Biomedicine, University of Aarhus, Aarhus, Denmark

**Keywords:** CSF, CSF Flow, Protein dynamics, Blood-derived proteins, Brain-derived proteins, Leptomeninges, Mannan binding lectin, Innate immune system in CNS, Blood- CSF barrier function

## Abstract

**Background:**

Mannan-binding lectin (MBL), a protein of the innate immune response is attracting increasing clinical interest, in particularly in relation to its deficiency. Due to its involvement in brain diseases, identifying the source of MBL in CSF is important. Analysis of cerebrospinal fluid (CSF) can provide data that discriminates between blood-, brain-, and leptomeninges-derived proteins. To detect the source of MBL in CSF we need to consider three variables: the molecular size-dependent concentration gradient between CSF and blood, the variation in transfer between blood and CSF, and the CSF MBL concentration correlation with the albumin CSF/serum quotient (QAlb), i.e., with CSF flow rate.

**Methods:**

MBL was assayed in samples of CSF and serum with an ELISA, coated with anti MBL antibodies. Routine parameters such as albumin-, immunoglobulin- CSF/serum quotients, oligoclonal IgG and cell count were used to characterize the patient groups. Groups comprised firstly, control patients without organic brain disease with normal CSF and normal barrier function and secondly, patients without inflammatory diseases but with increased QAlb, i.e. with a blood CSF barrier dysfunction.

**Results:**

MBL concentration in CSF was at least five-fold higher than expected for a molecular-size-dependent passage from blood. Secondly, in a QIgM/QAlb quotient diagram (Reibergram) 9/13 cases showed an intrathecal fraction in some cases over 80% of total CSF MBL concentration 3) The smaller inter-individual variation of MBL concentrations in CSF of the control group (CV = 66%) compared to the MBL concentrations in serum (CV = 146%) indicate an independent source of MBL in CSF. 4) The absolute MBL concentration in CSF increases with increasing QAlb. Among brain-derived proteins in CSF only the leptomeningeal proteins showed a (linear) increase with decreasing CSF flow rate, neuronal and glial proteins are invariant to changes of QAlb.

**Conclusions:**

MBL in CSF is predominantly brain-derived and all results pointed to the leptomeningeal cells as the source of the protein. The evaluation of this protein requires the interpretation of its absolute concentrations in CSF as a function of the albumin quotient, QAlb. This recognition of MBL in brain cells opens a new field of discussion about the function of the innate immune response in CNS in cases of acute and chronic neurological diseases.

## Background

### Blood-CSF barriers and CSF flow rate

Increased protein concentrations in the cerebrospinal fluid (CSF) of patients with neurological diseases, frequently ascribed to a blood-CSF barrier dysfunction, are due to pathologically-reduced CSF flow rates [1]. This view is based on the molecular diffusion/CSF flow theory [1] which shows that the concentration of a blood-derived protein in CSF is in equilibrium between the rate of diffusion into CSF and rate of elimination by CSF flow. The molecular size-dependent rate of diffusion is represented by the CSF/serum concentration quotients of the purely blood-derived proteins in normal CSF.

Albumin in CSF is derived exclusively from blood even in all kinds of pathological processes of neurological disease [1,2]. Therefore albumin became the generally accepted reference for the individual barrier function for blood-derived proteins (such as IgG, or IgM) in the form of the CSF/serum concentration quotient, QAlb [3]. As there are no transport systems for the passage for proteins from blood to CSF, the CSF/serum quotient of a blood-derived fraction of any protein molecule for which we know the molecular size can be estimated [1,4]. With this concept, which shows that a blood-CSF barrier dysfunction is not any kind of “leakage” at capillary structures but a consequence of the pathologically-reduced CSF flow rate, a change in QAlb can be interpreted as a change in CSF flow rate [1,4]. With this much wider view of the barrier function for proteins, it is necessary to extend our view from the blood-derived proteins to all proteins in CSF. But the influence of reduced CSF flow rate on proteins in CSF depends critically on the source of the proteins.

### Sources of CSF proteins

Three sources for CSF proteins can be identified. These are: firstly, blood-derived proteins in CSF (80% of total protein [2]) evaluated as CSF/serum quotients with reference to the albumin CSF/serum concentration quotient, QAlb (e.g. IgG, IgA, and IgM) [2,3]. The non linear reference range for blood-derived proteins in CSF forms the base for Reibergrams [2,3,5], which enables the sensitive and quantitative detection of additional intrathecal synthesis [3]. Secondly, brain cell- derived proteins are interpreted by their absolute concentration in CSF without reference to QAlb, since they are independent of CSF flow rate (e.g. Tau protein, S-100 B, neuron-specific enolase [4,6]). Thirdly, proteins are released from leptomeningeal cells into CSF. These are also evaluated as absolute CSF concentration but with additional reference to QAlb, because they have a linear correlation with CSF flow rate (e.g. beta trace protein, cystatin C [4,6]).

Inappropriate referencing of brain-derived proteins to their serum concentration using the CSF/serum quotient instead of the absolute CSF concentration leads to a loss of sensitivity for discriminations between groups, i.e., eventually to false interpretations of CSF data and disease pathologies. These different dynamics in CSF have been shown empirically and were derived quantitatively from the molecular diffusion/CSF flow model of Reiber [1,4,6]. This mathematical derivation from the laws of diffusion also explains the rostro-caudal concentration gradients which increase for blood-derived and decrease for brain cell-derived proteins in CSF, again with a particular variation for leptomeningeal proteins [4,6]. From these considerations it is possible to discriminate between the different sources of CSF proteins for diagnostic purposes. As a particular analytical tool for discrimination between blood- and brain-derived proteins, we have used in this evaluation the variation propagation, expressed as the coefficient of variation for the inter-individual variation between blood and CSF concentrations in a control group of patients.

### Mannan-binding lectin (MBL)

The increasing awareness for the innate immune system and its possible role in the CNS is documented in recent publications (cited in [7,8]): Mannan-binding lectin (MBL) [7] is of increasing clinical interest, since MBL deficiency [8] was found to be associated with different diseases, including infections, in systemic as well as neurological diseases [8-11]. MBL, a collagenous serum lectin (collectin), synthesized in the liver is believed to play an important role in innate immunity. The innate immune system is non-clonal and recognizes conserved patterns (pathogen-associated molecular patterns) on e.g. bacteria and yeast by genetically-encoded pattern recognition molecules. MBL thus recognizes patterns of mannose, glucose and N-acetyl-glucosamine residues on microbial cell walls. The binding of MBL to suitably spaced patterns of glycosylation initiates the activation of the complement cascade through MBL-associated serine proteases (MASPs) [7,12,13].

In previous studies MBL has been detected in CSF [10,14]. In this study, we evaluated the possibility that CSF MBL originates from within the brain, using data from two patient groups: a control group with normal QAlb, i.e., normal blood-CSF barrier function, and a second group with increased QAlb, but without cerebral inflammation.

## Methods

### Patients

**Figure 1:**
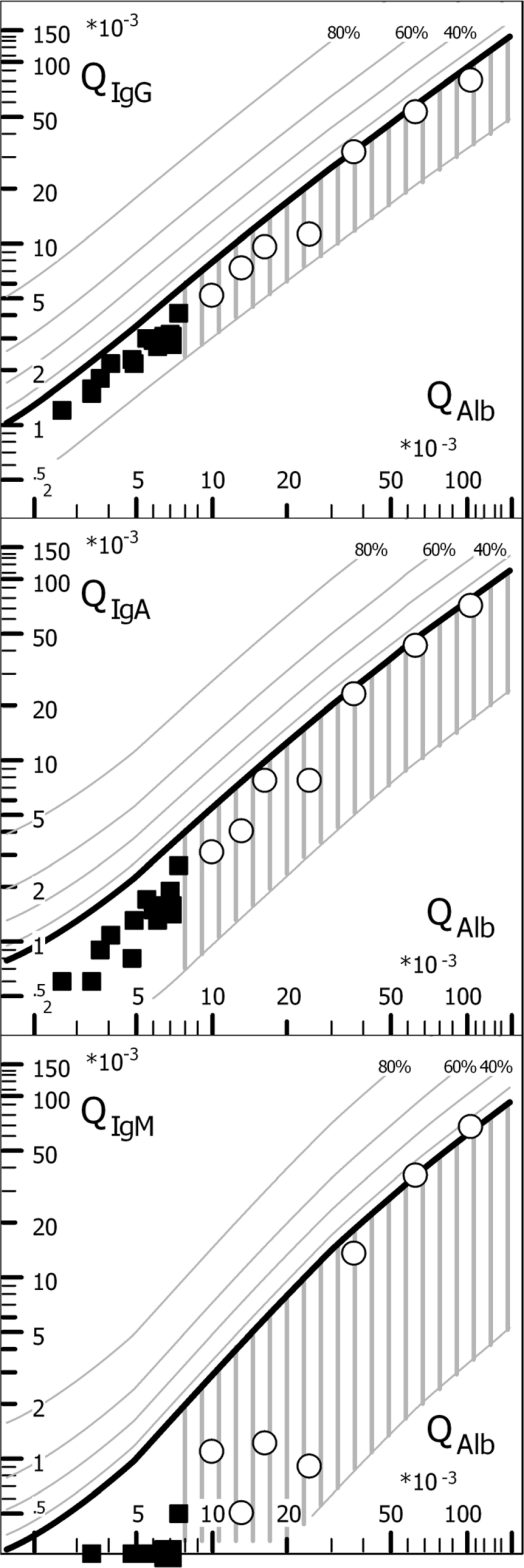
**Immunoglobulin and Albumin quotient diagrams (Reibergrams) of the normal controls (filled squares) and patients with blood-CSF barrier dysfunctions without an intrathecal immune response (open circles).** Oligoclonal IgG was absent in all patients, also in the one patient with a borderline IgM quotient. The age-related reference range for normal QAlb values (normal blood-CSF barrier function) was calculated according to the formula QAlb < (4+ age (years)/15) × 10^-3^. An intrathecal synthesis of IgG, IgA and IgM is indicated by quotient values above the hyperbolic upper limit curve, Qlim (bold line). The reference range for the blood–derived fractions in CSF is derived from data of 4300 patients (mean ± 3SD [1], corresponding to 99% probability of intrathecal synthesis if QIg > Qlim). The dashed lines above Qlim indicate the extent of the intrathecally synthesized fractions in% with reference to Qlim as 0% [3]. These diagrams are developed for maximal specificity for intrathecal synthesis with the Qmean + 3SD. For statistical comparison of mean values of groups the calculation refers to Qmean and for counting the frequencies of intrathecal fractions of the group we refer to Qmean + 2 SD (CSF Statistics Tool [15], with which also the diagram was created).

Lumbar CSF and serum samples originated from patients of the Department of the Neurological University Hospital, Goettingen. All samples were taken for routine analysis [2,3], indicated by diagnostic criteria with the informed consent of the patients. As no experiments with patients were involved, we did not ask for ethical approval. Nevertheless, after routine analysis, residual CSF and serum samples, stored throughout at 4°C, were made anonymous according to the general advice of the ethics committee of the Medical Faculty, University Goettingen. From these samples we selected retrospectively two groups for this study: a) Normal controls (N = 13) and b) Cases of barrier dysfunctions without intrathecal immune response (N = 7). Control patients were referred for lumbar puncture on clinical indications, but were determined to be normal with no indication of organic brain disease according to clinical and imaging criteria, e.g. tension headache or non-inflammatory polyneuropathies, and according to their CSF and blood data (normal CSF leukocyte count and protein values, no oligoclonal IgG, age-related normal albumin quotient, normal blood leukocytes and serum C-reactive protein, CRP). The immunoglobulin data are shown in the quotient diagrams (Figure 1, Reibergrams).

Patients with non-inflammatory diseases but with blood–CSF barrier dysfunction had increased CSF/serum albumin quotients (QAlb) as well as all other blood-derived CSF/serum protein quotients (QIgG, QIgA, QIgM) but without any intrathecal contribution to the CSF values, as shown in the quotient diagrams (Figure 1, Reibergrams). Oligoclonal IgG in CSF and intrathecal IgG, IgA or IgM synthesis were exclusion criteria. Patients in this group typically had spinal canal stenosis, spinal tumor or disc prolapse. They had normal CSF cell counts and typical findings in electromyography and magnetic resonance tomography of the spine.

### Routine analysis of CSF

Analysis of albumin, immunoglobulins IgG, IgA, IgM in CSF and serum, oligoclonal IgG and cell count was performed as described earlier [2,3]. The age-related reference range for increased albumin quotients (blood/CSF barrier dysfunction) was calculated according to QAlb > (4 + Age(y)/15) × 10^-3^[3].

### Quantification of MBL in serum and CSF

The concentration of MBL in serum was estimated via its lectin activity measured with europium-labeled anti-MBL antibody. A detailed description of buffers and reagents has been given elsewhere [16]. In brief, monoclonal anti-MBL antibody was coated onto the surface of microtiter wells. Plasma or serum samples, diluted 1:100 in a high ionic strength buffer (preventing coagulation), calcium (needed for the binding of MBL), HSA (w/v) and heat-aggregated normal human IgG, was added to the wells. Following incubation the wells were washed and europium-labeled anti-MBL antibody was added. After another incubation and wash, enhancement buffer was added and the bound europium was measured by time-resolved fluorometry. Dilutions of standard serum as well as samples of serum with known high (1046 ng MBL/ml), middle (251 ng MBL/ml) and low (38 ng MBL/ml) concentrations were included as internal controls. The inter- assay coefficients of variation (CV), calculated on 20 assays, were 8% and 11% respectively. The concentration of CSF MBL levels was measured by the same procedure as serum but using undiluted CSF samples.

### Statistics for the CSF/serum quotients

The CSF Statistics Tool was used, based on nonlinear reference of the protein CSF/serum quotients to the individual albumin quotient [15]. For details see also legend of Figure 1. Statistics for the comparison of inter-individual CV values were not used as there was no data overlap of these groups.

## Results

### Protein concentrations

**Figure 2:**
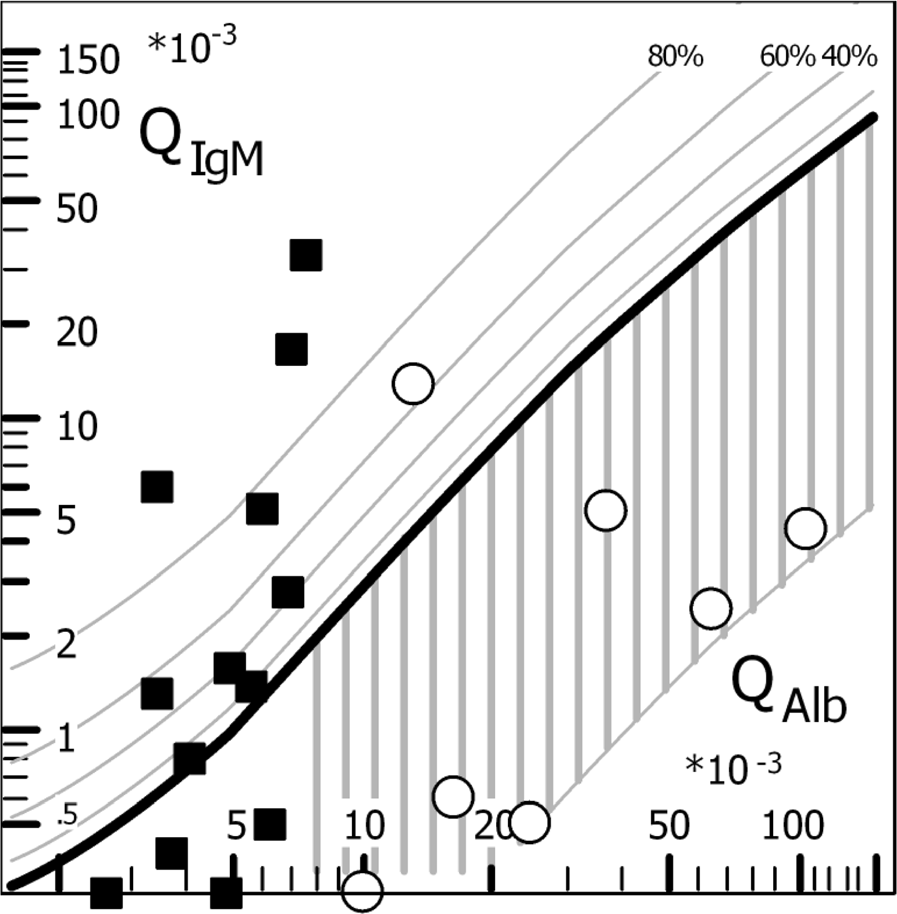
**Mannan-binding lectin (MBL) data in an IgM Reibergram.** The CSF/serum quotients QMBL are shown as a function of the albumin quotients, QAlb, in a diagram with the upper limit for the IgM molecule shown by the bold curve and with the reference range for the IgM molecule which has a similar molecular size as MBL. The filled squares represent the data of normal controls and the open circles of patients with a barrier dysfunction in the absence of an intrathecal immune response. 9/13 controls are obviously above the upper limit of the reference range, i.e., they have an intrathecal MBL fraction over 80% of total CSF MBL in addition to the blood-derived MBL fraction. The largest QMBL values correspond to the lowest serum MBL values (Table 1). For explanation of the diagram see legend to Figure 1 and [3]. The diagram was created with the CSF Statistics Tool [15].

The complete set of protein data for CSF and serum of both groups (i.e., with and without a blood CSF barrier dysfunction) is shown in Table 1. The corresponding CSF/serum concentration quotients of MBL (QMBL, Table 1) are shown as a function of the albumin quotient, QAlb (Figure 2). The QIgM/QAlb quotient diagram (Reibergram [2,3,5]) was used as a base for Figure 2 to get a reliable hyperbolic reference range for a theoretically blood-derived MBL fraction in CSF. The free MBL molecule is a similar size to IgM, the reference range of which was developed from a group of 4300 patients [1]. To obtain a reliable specific MBL diagram, a much larger data set would be required, but according to our results would have no diagnostic relevance. Nine out of thirteen patients in the control group showed QMBL values larger than the upper reference line Qlim (Figure 2, bold line), some with intrathecal fractions larger than 80% of the total MBL concentration in CSF. This clearly indicates that there is a dominant fraction of MBL in CSF that does not originate from blood.

**Table 1:** CSF/serum quotients for albumin, IgG, IgA, IgM and MBL with individual CSF and serum concentrations of MBL in controls and patients with barrier dysfunctions

	**Normal Blood CSF Barrier function**						
**Pat**	**Q Alb × 10**^**3**^	**Q IgG × 10**^**3**^	**Q IgA × 10**^**3**^	**Q IgM × 10**^**3**^	**Q MBL × 10**^**3**^	**MBL-CSF ng/ml**	**MBL-S ng/ml**
1	6,8	2.9	1.5	0.3	2,77	1,1	397
2	3,4	1.6	0.6	0.1	1,33	1,31	979
3	6,0	2.9	1.5	<0.3	5,21	1,96	376
4	3,7	1.8	0.9	<0.2	0,39	0,2	514
5	7,0	3.2	1.9	0.3	16,7	0,2	12
6	4,9	2.3	0.8	0.2	0,34	1,58	4598
7	5,0	2.2	1.3	0.2	1,58	1,15	728
8	6,2	2.7	1.3	<0.3	0,53	0,11	207
9	2,6	1.2	0.6	<0.1	0,27	0,27	985
10	5,6	3.0	1.7	0.3	1,39	0,82	589
11	3,4	1.5	0.6	0.1	6,00	1,82	303
12	4,1	2.2	1.1	<0.3	0,77	0,7	913
13	7,5	4.2	2.6	0.5	33,8	1,15	34
**Blood CSF barrier dysfunction**							
14	13.4	7.2	4.0	0.5	12.75	0.88	69
15	10.3	5.2	3.1	1.1	0.61	1.14	6784
16	16.6	9.6	7.7	1.2	0.59	1.59	2678
17	36.7	32.2	22.8	13.6	5.00	6.79	1346
18	64.6	52.5	42.4	36.6	2.41	4.19	1739
19	24.8	11.3	7.6	0.9	0.64	2.16	3391
20	106	79.7	70.2	67.9	4.39	8.43	1921

The control group (upper part) represents patients with normal CSF data and normal barrier function. The lower part represents the group of patients with a blood-CSF barrier dysfunction without signs of an inflammation in brain.

### Data analysis

From the statistics in Table 2 we get further systematic information:

**Table 2:** **Coefficients of variation (CV) of MBL data from the control group with age-related albumin quotients in Table**1**(median of n = 13)**

	**QAlb×10**^**3**^	**QMBL×10**^**3**^	**CSF-MBLng/ml**	**Serum MBLng/ml**
Median (n = 13)	5.0	1.39	1.1	514
Mean	5.1	0,73	0.95	818
SD	1.5	0,59	0.63	1183
CV (%)	30	**81**	**66**	146

For MBL with a molecular weight of 6 × 96 kDa = 576 kDa, the mean transfer gradient was similar to IgM. i.e., 3000:1 [1]. This would mean that with a median concentration of about 500 ng/ml in blood samples from the control group (Table 2), a blood –derived mean CSF concentration of MBL of < 0.2 ng/ml would be expected. In fact, there was a median concentration > 1 ng/ml, which is 5 fold larger than expected for a blood-derived fraction. This is indicative for a predominantly brain-derived MBL fraction (> 80%) in CSF.

The biological variability, expressed as the coefficient of variation (CV), for MBL in CSF is much smaller (CV = 66%) than that of the serum MBL value (CV = 146%). In the case of a blood-derived protein in CSF, an increased coefficient of variation would be expected, due to the additional biological variation originating from the individual barrier function. These consequences of the data in Table 2 mean that the CSF concentrations of MBL were not, or only negligibly, correlated with the serum concentrations. Therefore MBL in CSF is primarily a brain-derived protein, i.e., it must be evaluated on the base of its absolute concentration in CSF [4,6].

### CSF flow-related MBL concentration in CSF

**Figure 3:**
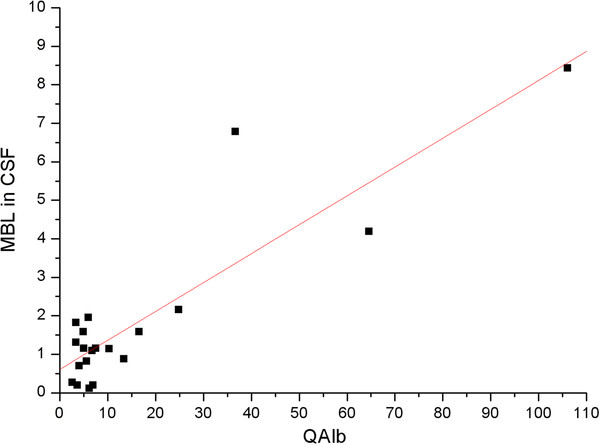
**CSF MBL concentration in lumbar CSF as a function of the albumin quotient, QAlb.** The regression line between CSF MBL (ng/ml) and Q Alb × 10^3^ refers to the data of all patients, the normal control group and the group with blood-CSF barrier dysfunctions, with the function: CSF MBL(ng/ml) = 0.61(ng/ml) + 0.075 QAlb.

The correlation of the absolute CSF concentration of MBL with the albumin CSF/serum quotient, QAlb, is shown in Figure 3 for all patients without and with a barrier dysfunction (Table 1). This diagram shows that the MBL concentration in lumbar CSF increased with increasing QAlb. This is a specific result, as only the leptomeningeal proteins show such a dynamic, in contrast to neuronal or glial proteins the concentrations of which are invariant to the increase of QAlb, i.e., the decrease of CSF flow rate. In combination with the results described above, there is no other interpretation available than that MBL in lumbar CSF is a protein predominantly derived from the leptomeninges. This result is quantitatively explained by the theoretical model for brain-derived proteins in CSF [1,4] and in this particular case supported by morphological findings [17,18].

## Discussion

### Sensitivity of MBL assays

The sufficiently sensitive analysis of MBL in CSF represents an analytical challenge [16]. For the analysis of MBL in CSF it is important to use an ELISA coated with MBL antibodies which has a higher affinity for MBL compared to a mannan-coated ELISA.

### Blood-CSF barrier function for MBL

In fact, we do not know the effective molecular size of MBL, relevant for its passage from blood to CSF. The blood-derived MBL fraction in CSF may be much smaller than calculated on the base of the molecular size of free MBL (results). It is possible that MBL with its tendency to bind other proteins does not pass the blood CSF barriers as a free MBL molecule but as a larger MBL-MASP complex, i.e., with bound serine proteases [12,13]. If this were the case, it would mean that much less than 20% of MBL in normal CSF originates from blood.

### The intrathecal fraction of MBL

The intrathecal MBL fractions are shown directly in the IgM quotient diagram in Figure 2. Due to its similar molecular size to MBL, the IgM diagram was used [1,3] for a hypothetical evaluation of MBL data. On this basis from Figure 2 it was concluded that MBL in CSF has a large intrathecally synthesized fraction in most cases (9/13 cases). As shown in Table 1 the largest QMBL values in Figure 2 were associated with the smallest serum values. This indicates that the CSF and serum values have no biological correlation, i.e. the calculation of a quotient is just a virtual number without a physical reality. There is a second bias in the calculation of an intrathecal fraction with reference to the reference range in Figure 2 for the molecular size of the free MBL molecule. The reference line in Figure 2 would be lower if MBL passes the barriers as a larger MBL-MASP complex, with the consequence of a larger intrathecal fraction.

### The coefficient of variation and the variation propagation

It may be a surprising application of the coefficient of variation (CV) to characterize the connection of a parameter in two different compartments of a biological system.

If two independent functions contribute to the final result in mathematics this is treated as error propagation. The variability of each function adds up to a common larger variability. In physiology of protein dynamics this means that the variability of a protein concentration in blood samples of a group of persons is connected to the independent variability of their individual barrier functions (CSF flow rate, body length, CSF pressure, etc.) additionally influencing the protein concentration in CSF. Due to such variation propagation there is a larger variation in the albumin concentration in CSF samples (CV = 26%) compared to the corresponding variation in blood samples (CV = 15%) in a control group (N = 20, mean age of 46 years, mean QAlb = 5.6 x10^-3^). Of course there are different options in a biological system to get such a result, but if the inverse combination with a smaller or still equal variation in CSF compared to serum samples of the same group is obtained, it can be concluded that there is no diffusion-dependent association of the parameter in the different compartments. But this is exactly what was obtained for MBL (Table 2): The coefficient of variation of MBL in CSF (CV = 66%) was much smaller than the serum variability in this group (CV = 146%). In spite of the small number, this value is relevant as a similar result was obtained from the data of a much larger group of blood donors (N = 350) with a median of 1100 ng/ml and a coefficient of variation CV = 128%.

So the CV data for MBL lead to the conclusion that the MBL concentration in CSF does not vary in concordance with the serum concentration (also obvious in Table 1). This means that the CSF concentration of MBL is derived predominantly from brain and follows the dynamics of brain-derived proteins.

### Leptomeningeal proteins in CSF

Proteins released from leptomeningeal cells into CSF (not by intercellular passage from blood as blood-derived proteins) accumulate in CSF with decreasing turnover rate, i.e. slower CSF flow rate. Correspondingly their concentration correlates with QAlb. As shown for beta trace protein the correlation with QAlb is linear, as expected from the theory [4]. Figure 3 shows the corresponding approach by which the absolute MBL CSF concentration is shown as a function of the albumin quotient. The result clearly indicates a dynamic typical for a leptomeningeal protein. For testing the linearity for the relation between CSF MBL and QAlb we would need a larger number of data, but already with this small number it is clear that the CSF MBL concentration is increasing with increasing QAlb, i.e. the MBL concentration is definitely not invariant to the changing CSF flow rate.

As a consequence of empirical data and theory [4] the rostro-caudal gradient of leptomeningeal proteins should increase, i.e., an increasing concentration of MBL between ventricular, cisternal and lumbar CSF could be anticipated in the normal person. Such data would be useful confirm or refute the above conclusions for MBL.

An important confirmation of our conclusions that MBL is a leptomeningeal protein comes from histochemical investigations in animals [17,18] which show directly the presence of MBL in the leptomeninges.

Hence, the following arguments are found for MBL in CSF as a predominantly leptomeningeal protein:

1. The CSF concentration of MBL is larger than expected from a corresponding molecular size-dependent passage from blood.

2. An intrathecal MBL fraction in CSF is observed in more than 80% of the controls.

3. The smaller variability in CSF compared to blood concentrations of MBL, i.e. the absence of variation propagation, clearly excludes blood as the primary source for the MBL concentration in CSF.

4. The dynamics of the absolute MBL concentration in CSF as a (linear) function of QAlb point to a leptomeningeal protein.

5. Morphological data from animals show that MBL can be present in the leptomeninges.

Using established methods for evaluation of CSF data [2,3,5,15], a theoretically-founded, physiologically-relevant interpretation of the CSF flow-related dynamics of MBL in CSF has been obtained for the first time and the consequences for clinically relevant data evaluation can be identified.

## Conclusions

This study has shown that mannan-binding lectin in CSF is predominantly brain-derived, most likely from the leptomeningeal cells. There is a negligible physiological connection between the MBL fractions in CSF and serum. The evaluation of the clinical role of this protein requires the measurement of absolute concentration in CSF as a function of the CSF/serum albumin quotient. The recognition that MBL originates in brain cells opens a new field of discussion about the function of the innate immune response in CNS in acute and chronic neurological diseases.

## Abbreviations

MASP: MBL-associated serine protease; MBL: Mannan-binding lectin; QAlb, QIgG, QIgA, QIgM, QMBL: CSF/serum concentration quotient of albumin, IgG, IgA, IgM, MBL correspondingly; Qlim: Upper limit of the reference range for blood-derived proteins in CSF.

## Competing interests

There are no competing interests for any of the authors.

## Authors’ contributions

H R: Sample acquisition, theoretical interpretation of data. B P-D: Supervisor for clinical interpretation of MBL, coordination of the patient data acquisition. J C J: Laboratory analysis of MBL in CSF and serum. A J D-C: Organizer of the study, immunological interpretation of MBL data. All authors have read and approved the final version of the manuscript.
